# Recent Advances in Dysuricemia: Toward Optimal Serum Urate Level

**DOI:** 10.3390/biomedicines12051094

**Published:** 2024-05-15

**Authors:** Masafumi Kurajoh, Akiyoshi Nakayama

**Affiliations:** 1 Department of Metabolism, Endocrinology and Molecular Medicine, Graduate School of Medicine, Osaka Metropolitan University, Osaka 545-8585, Japan; 2 Department of Integrative Physiology and Bio-Nano Medicine, National Defense Medical College, Tokorozawa 359-8513, Japan

## 1. Introduction

We are pleased to present the Special Issue “Dysuricemia: Recent Advances in Urate Research from Hypouricemia to Hyperuricemia/Gout”. This collection of five research articles and four reviews addresses various topics in this research area, including the novel disease concept of “dysuricemia”, which has been proposed to describe disorders of urate handling and/or metabolism via xanthine oxidoreductase (XOR), and to interpret the spectrum from hypouricemia to hyperuricemia/gout as a single disease category. Here, we summarize the contributions of these nine articles to conclude this pioneering collection of works on dysuricemia.

## 2. Published Articles

Uric acid is much more than just a metabolic waste product; it can also acquire antioxidant properties by scavenging reactive oxygen species (ROS) and pro-oxidant properties by generating ROS [1,2,3,4]. Kurajoh et al. demonstrated that low uric acid levels are associated with higher rates of severe COVID-19 progression. However, uric acid levels are inversely associated with C-reactive protein (CRP) levels, and the association between the level of uric acid and severe COVID-19 progression differs significantly both with and without the inclusion of CRP levels. Low uric acid may contribute to severe COVID-19 progression via increased inflammation in subjects who do not exhibit signs of hyperuricemia [5]. This indicates that heritable hypouricemia, such as renal hypouricemia and xanthinuria, may be a risk factor for severe COVID-19; further studies are needed to elucidate this relationship.

Uric acid is mainly produced by XOR in the human liver [6,7]. To accurately evaluate XOR activity in liver disease, Sato et al. measured the plasma XOR activity in patients with liver disease using a novel, sensitive, and accurate assay that combines liquid chromatography and triple quadrupole mass spectrometry to detect [^13^C_2_, ^15^N_2_] uric acid using [^13^C_2_, ^15^N_2_] xanthine as a substrate [8,9]. They found that plasma XOR activity is generally higher in liver disease and is closely correlated with liver test parameters, especially serum ALT levels, regardless of etiology or plasma xanthine levels. Plasma XOR activity might thus reflect the active phase in various liver diseases [10]. This study suggests that XOR may play a role in the organ damage that occurs during liver disease; however, further research is needed to clarify its significance.

Gout is caused by prolonged asymptomatic hyperuricemia, and the rates of prevalence of asymptomatic hyperuricemia and gout are increasing worldwide [11,12,13]. To identify potential biomarkers that can differentiate gout from asymptomatic hyperuricemia, Ohashi et al. conducted a genetic analysis of urate transporters and metabolomic analysis as a proof-of-concept study. They found that although urate transporters play a critical role in elevating serum urate levels and promoting hyperuricemia, the progression from asymptomatic hyperuricemia to gout might be closely related to other genetic and/or environmental factors that affect carbohydrate metabolism and urinary urate excretion [14]. Interestingly, hyperuricemia is necessary, but might not be sufficient, to cause gout. This finding could lead to novel therapeutic concepts for preventing asymptomatic hyperuricemic patients from developing gout by handling factors that affect carbohydrate metabolism and urinary urate excretion. 

XOR inhibitors reduce serum urate levels and are pivotal therapeutic agents for treating gout and hyperuricemia. Recent research has revealed its relationship with other diseases. Three research articles and one review that address this are also included in this Special Issue.

The protective effect of uric acid-lowering therapy against cardiac diseases remains controversial. Fujishima et al. found that treatment with topiroxostat, a non-purine selective inhibitor of XOR [15], improved arterial stiffness parameters—that is, the cardio–ankle vascular index and brachial–ankle pulse wave velocity—in hyperuricemic subjects with higher baseline ALT levels. They also demonstrated that it was accompanied by significant suppression of increased plasma XOR activity. These results suggest that that topiroxostat has therapeutic potential for improving arterial stiffness and preventing atherosclerotic disease in patients with liver dysfunction [16]. These studies also indicate that plasma XOR levels are higher in liver dysfunction and that treatment with topiroxostat may limit the contribution of increased XOR to cardiovascular disease.

Smoking and hyperuricemia have been independently reported to be associated with chronic kidney disease (CKD) [17,18,19]. While investigating the effect of a combination of these risks with renal arteriolosclerosis in IgA nephropathy patients, Shinzato et al. discovered that smoking affected renal arteriolar wall thickening in the presence of hyperuricemia, but not in the absence of hyperuricemia in patients with IgA nephropathy. These findings suggest that a combination of smoking and hyperuricemia may promote the progression of CKD by enhancing renal arteriolosclerosis [20]. Hyperuricemia may have an additive or synergistic effect on vascular lesions caused by cardiovascular risks such as smoking. 

Kotozaki et al. reviewed recent progress in our understanding of the evidence linking human plasma XOR activity to cardiovascular disease (CVD). Plasma XOR activity has been proposed as a biomarker that can be used to assess metabolism, renal function, and CVD progression [21,22,23]. Plasma XOR activity has also been proposed as a possible risk factor for CVD. However, the authors believe that more research is needed to gain an understanding of the mechanisms of plasma XOR activity and the development of CVD [24].

Miake et al. reviewed the impact of dysuricemia on kidney function, including urolithiasis, renal tubular damage, and kidney injury. The underlying mechanisms may be the result of two types of endothelial dysfunction: one induced by intracellular UA, monosodium urate and XOR under hyperuricemic conditions [25,26], and the other by a depletion of nitric oxide and endothelium-derived highly polarized factors under hypouricemic conditions [27]. They concluded that urate-lowering agents should be recommended for the treatment of kidney disease in hyperuricemic patients, and that XOR inhibitors might also be useful for reducing oxidative stress in renal hypouricemia patients, in addition to hydration and urinary alkalization [28].

As described here, uric acid has a dual nature in the human body. Nakayama et al. [29] and Otani et al. [30] presented a new disease concept referred to as “dysuricemia” based on the antioxidant and oxidant-promoting effects of uric acid [1,2,3,4]. Otani et al. revealed that both hypo- and hyperuricemia are involved in the pathogenesis of a number of common diseases, including lifestyle-related diseases, renal dysfunction, cardiovascular events, neurological disorders, and gout. Nakayama et al. illustrated this concept using a Figure in which three typical patterns of disease risk associated with serum urate level are represented: the “gout pattern”, the “CKD and CVD pattern”, and the “neurodegenerative pattern”. Both reviews stress the importance of maintaining normouricemia, or optimal serum urate level, to prevent these common diseases. The concept of “dysuricemia” should therefore enable the spectrum from hypouricemia to hyperuricemia/gout to be interpreted as a single disease category and will encourage researchers to focus more closely on the dual nature of uric acid.

## 3. Conclusions

Elevated serum urate levels are unique to humans, due to a lack of urate oxidase seen during our process of evolution from apes [31]. As shown here, there are still many research and clinical questions pertaining to urate and urate-related diseases that have yet to be satisfactorily addressed. We believe that the recent advances in dysuricemia research covered in the present Special Issue will uncover many new roles of urate. We hope that, in the near future, the disease burden exerted by common diseases such as gout, CKD, CVD, and neurological disorders, can be reduced by maintaining optimal serum urate levels via the prevention of hyperuricemia and hypouricemia, which we now collectively refer to as dysuricemia.
